# Developmental dynamics of early reading skill, literacy interest and readers’ self-concept within the first year of formal schooling

**DOI:** 10.1007/s11145-018-9843-8

**Published:** 2018-04-17

**Authors:** Bente Rigmor Walgermo, Njål Foldnes, Per Henning Uppstad, Oddny Judith Solheim

**Affiliations:** 1 Norwegian Reading Centre, University of Stavanger, P.O. Box 8600 Forus, 4036 Stavanger, Norway; 2 BI Norwegian Business School Stavanger, Hesbygaten 4, 4014 Stavanger, Norway

**Keywords:** Early reading skills, Literacy interest, Reader self-concept, Cross-lagged modeling within first grade

## Abstract

Previous studies have documented robust relationships between emergent literacy and later reading performance. A growing body of research has also reported associations between motivational factors and reading in early phases of reading development. However, there is less research about cross-lagged relationships between motivational factors and reading skills in beginning readers. To examine relationships between early reading skills, literacy interest and reader self-concept, we tested 1141 children twice during their first year of formal reading instruction in school. Cross-lagged analysis showed strong stability in reading skills and medium stability in literacy interest and reader self-concept over the first school year. We also found bidirectional relationships between reading skills and self-concept and between the motivational components of literacy interest and reader self-concept. In the final part of the article, we address the potential theoretical progress attainable through the use of cross-lagged designs in this field.

## Introduction

Children who are driven by interest are likely to devote more time and effort to reading tasks and often feel more competent as readers (Ecalle, Magnan & Gibert, 2006). Students who see themselves as good readers will anticipate success in academic situations and often perform better at academic tasks than students who exhibit poor self-belief and hence anticipate failure (Murphy & Alexander, 2000; Zimmerman, 2000). Thus, both literacy interest and reader self-concept are considered important for students’ learning and for the development of reading skills. The state of the art in motivation research suggests that reading skills, literacy interest and reader self-concept develop in an interrelated way. Previous studies have documented bidirectional associations between motivational beliefs and reading performance in middle- and secondary-school readers (Conradi, Jang & McKenna, 2014; Petscher, 2010; Schiefele, Schaffner, Möller & Wigfield, 2012). However, we know less about how early these mechanisms come into play, because few studies have investigated the associations between motivation and reading performance in young readers. In addition, most previous research in this field has relied on cross-sectional designs (e.g., Chapman, Tunmer & Prochnow, 2000; Morgan & Fuchs, 2007). To the best of our knowledge, our study is the first to use a latent-variable longitudinal cross-lagged analysis to investigate the associations between interest, self-concept and skill in the first year of formal schooling. The findings from our study, with its particular time window, illustrate the dynamic interplay between motivation and skill.

### Motivation and skill development

In our approach, interest is conceptualized as an affective state and centers on students’ subjective experience of literacy situations. We focus on students’ enjoyment of literacy activities in school and at home because we are concerned with the feelings that students bring with them into learning situations—feelings that we assume will exert an influence on their performance. Along these lines, we adopted Ainley’s (2006) definition of “interest”: “Interest is conceptualized as an affective state that represents students’ subjective experience of learning; the state that arises from either situational triggers or a well-developed individual interest” (Ainley, 2006, p. 392). We take care to recognize that “learning” with in the lines of this definition, does not only involve formal instruction, but also includes developmentally-relevant literacy experiences that children have outside of the school context.

Students’ perceptions of their own competence in relation to academic achievement have been conceptualized in different ways—with self-efficacy and self-concept being the two most widely studied constructs (Pajares & Schunk, 2001). Self-efficacy in academic settings relates to more task-specific beliefs (e.g., knowing the letters in one’s own name), whereas self-concept refers more generally to beliefs about one’s competence and ability (e.g., expecting to be able to learn how to read). Past research has shown that a person’s self-concept is formed by previous experience, especially by reinforcement from significant others (Shavelson, Hubner & Stanton, 1976). In addition, the frames of reference or standards by which we judge our accomplishments and compare ourselves with other people are a powerful influence on the formation of self-concept (Bong & Skaalvik, 2003). Similarly, children’s literacy interest and their later engagement in reading situations are also strongly influenced by their literacy experiences at home as well as in kindergarten and school (Mata, 2011; Sonnenschein & Munsterman, 2002).

It has been assumed that students’ ability-related self-concept provides a basis for their task motivation (i.e., their interest; Deci & Ryan, 1987; Eccles, 1983; Wigfield & Eccles, 2000). In a longitudinal study covering grades 1–12, Jacobs, Lanza, Osgood, Eccles and Wigfield (2002) found that children were much more likely to value mathematics, languages, arts, and sports highly if they felt competent in the domain in question. Also, Spinath and Spinath (2005) reported a concurrent correlation of .31 between first-graders’ general learning motivation (i.e., interest) and their self-belief. Results from these studies indicate that if someone feels competent at an activity, he or she is more likely to be interested and will value that activity, and also that, in learning situations, a person’s level of interest is related to his or her self-belief.

In addition to motivational components, several other factors are known to influence the development of reading skills. Children who acquire more than one language in early childhood often experience a delay in their language and literacy development (Fawcett, 2016). Further, the parents’ (especially the mother’s) level of education has been found to be related to students’ reading performance (Davis-Kean, 2005). Studies have also found gender differences with respect to motivation in young readers in that boys overall seem to value reading less than girls do in the early elementary years (Marinak & Gambrell, 2010). Consequently, gender, the mother’s educational level and the language(s) spoken at home have been chosen to function as covariates in our investigations of the first-grade dynamics of reading skills and motivation.

### Methodological considerations in the design of prior studies in the field

**Fig. 1:**
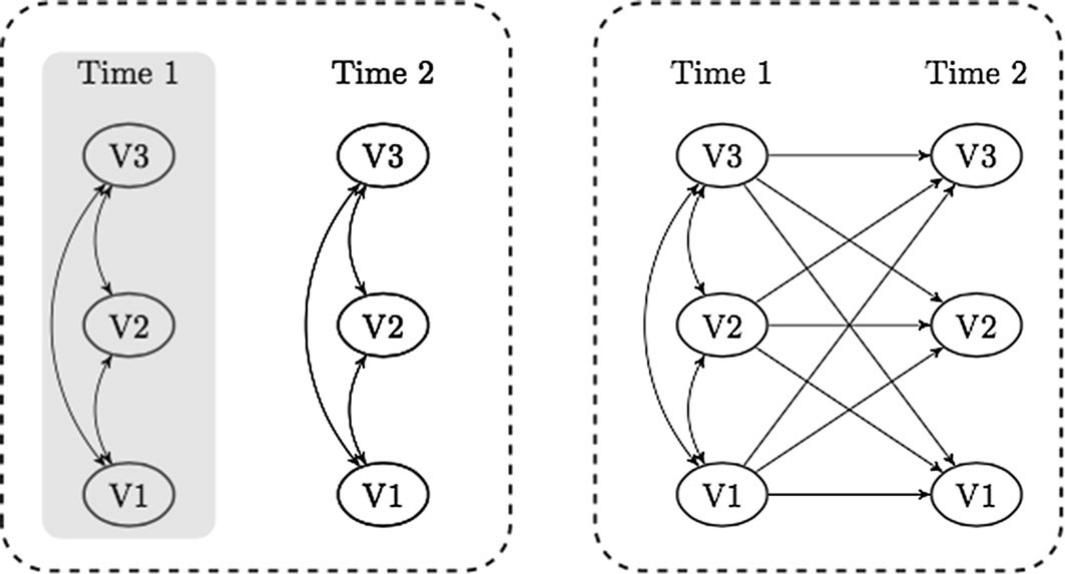
Three types of analysis of the relationships among variables V1, V2 and V3. Typically, V1 and V2 are measures of motivational constructs whereas V3 is a measure of reading skills. The first type, in the shaded box, only involves cross-sectional associations at one time point. The second type, depicted in the entire left-hand dashed rectangle, involves independent analysis of associations at Time 1 and Time 2. Finally, the third type, in the right-hand dashed rectangle, also encompasses cross-lagged and autoregressive effects

Previous studies addressing the relationship between reading skills and motivation for reading have used different designs, both in terms of the number of data-collection points and in terms of the methods used to analyze data. Broadly speaking, three methods of data collection and analysis have been employed (see Fig. 1).

First, as schematically shown by the shaded box in Fig. 1, some studies report findings from strictly cross-sectional data, obtaining associations at only a single time point. In a study by Baroody and Diamond (2016), literacy interest, as rated by the students’ teachers, was not associated with early reading skills in a sample of 167 5-year-olds. This is in line with findings reported by Walgermo, Frijters and Solheim (2018) that indicate no associations between children’s literacy interest and reading skills in the first weeks of formal schooling. Reader self-concept, on the other hand, has been found to be significantly associated with reading skills in previous studies involving first-grade students (see Morgan, Fuchs, Compton, Cordray & Fuchs, 2008; Walgermo et al., 2018).

Second, as sketched in the left-hand dashed rectangle in Fig. 1, some studies gather data at successive time points but restrict their analysis to cross-sections of the panel data. That is, associations among variables are investigated separately at each time point. Changes in these associations between time points are then described and commented upon without further statistical analysis. For instance, the correlation between interest and skill at the first time point is typically compared with the correlation between interest and skill at later time points, and the change in magnitude (a stronger or weaker correlation) is interpreted informally. Although this second method does yield some indications about the development of associations, there is no formal way of testing more specific hypotheses about reciprocal relationships among the variables under investigation—given that the initial levels of emergent literacy, interest and self-concept are not taken into account. Chapman and Tunmer (1997) found links between reader self-concept and skill 7 weeks into the first grade, and they went on to track changes in 60 students’ reading skill and self-concept over the first 3 years of school, finding that the correlation between reading skill and reader self-concept increased steadily over that period: from .11 over .21 to .35.

The third method of data analysis incorporates cross-lagged paths (i.e., as outlined in the right-hand dashed rectangle in Fig. 1). In these models, two or more variables are measured on two or more occasions, and interest is centered on the associations between the various variables over time. These models makes it possible to establish reciprocal relationships and to make claims about reciprocity across constructs and time points. So far, most longitudinal studies that have applied path analysis when investigating the relationship between motivational beliefs and reading skills have involved older students, in the fourth grade or above (e.g., Marsh, Trautwein, Lüdtke, Köller & Baumert, 2005).

The results from the few studies involving younger students are inconclusive. Viljaranta, Lerkkanen, Poikkeus, Aunola, and Nurmi (2009) investigated the cross-lagged relationship between literacy-related task motivation (i.e., how much a person enjoys or likes a certain school subject or task) and literacy performance in Finnish 5- and 6-year-olds in their final year of kindergarten, finding no statistically significant relationships. Nurmi and Aunola (2005) also failed to find any significant associations between self-concept, task motivation and reading achievement in Finnish first- and second-graders using mixed cross-sectional clustering with longitudinal analysis of state changes. Further, Chapman and Tunmer (1997) found that neither reading skill nor reader self-concept in the first grade directly affected each other in the second grade. However, they did find a significant path from second-grade reading to third-grade reader self-concept. Schiefele, Stutz and Schaffner (2016) also found a statistically significant reciprocal relationship between one component of intrinsic motivation—involvement—and reading comprehension in the same age group (i.e., second- and third-graders). Taken together, previous research indicates that the interactions between motivational beliefs and reading performance start to occur during the second or third year of schooling, and hence it might be assumed that experiences and feedback from learning to read in the context of formal reading instruction are a prerequisite in order for this interaction to appear.

The effect of motivational beliefs on achievement is hypothesized to be mediated by motivational behavior. If we extend the scope of our research review to include studies investigating the relationship between achievement strategies (i.e., students’ *behavior* when working on literacy tasks) and subsequent reading skills, we find that interactions have been observed at an earlier stage. Onatsu-Arvilommi and Nurmi (2000) and Aunola, Leskinen, Onatsu‐Arvilommi and Nurmi (2002) used cross-lagged designs on Finnish first-graders, finding that children who displayed a task-avoidant rather than a task-focused achievement strategy in school performed less well in reading later on. They also reported that a low level of reading skill at school entry predicted subsequent task avoidance after the second half of the first grade.

### Learning to read in the Norwegian school system

The present study was undertaken in a Norwegian school context. Formal reading instruction in Norway starts when children begin the first grade, which they do in August of the year of their sixth birthday. Before this, Norwegian children typically attend the *barnehage* (where they may be enrolled as early as at the age of 10 months). Approximately 95% of Norwegian children attend the *barnehage* full-time from 3 years of age (35 h weekly; NDET, 2013). In the *barnehage*, childhood is valued as an important life stage in and of itself, not just as a preparation for adulthood (OECD, 2013). Key principles governing activities there are *frilek* (free or undirected play) and *medvirkning* (participation, meaning that children are routinely consulted about decisions in their daily activities). Unlike in countries with a comprehensive system of early-childhood education, the Norwegian system does not aim for specific behavioral outcomes (OECD, 2013). Among other things, this means that literacy skills are promoted using different play-like methods and activities which are largely driven by the children’s own initiative (NDET, 2013). As a result of this, Norwegian children enter the first grade without any experience of formal reading instruction. This means that there is extreme variation in the emergent literacy skills of Norwegian school starters: some children have no experience at all with learning how to read, others know some or most letters, and some have acquired decoding reading skills and are able to read texts on their own. Against this background, it is particularly important in Norway to understand how the level of emergent literacy and motivation with which children enter school is related to their reading skills and the respective motivational component after 1 year of formal schooling.

### The present study

**Fig. 2:**
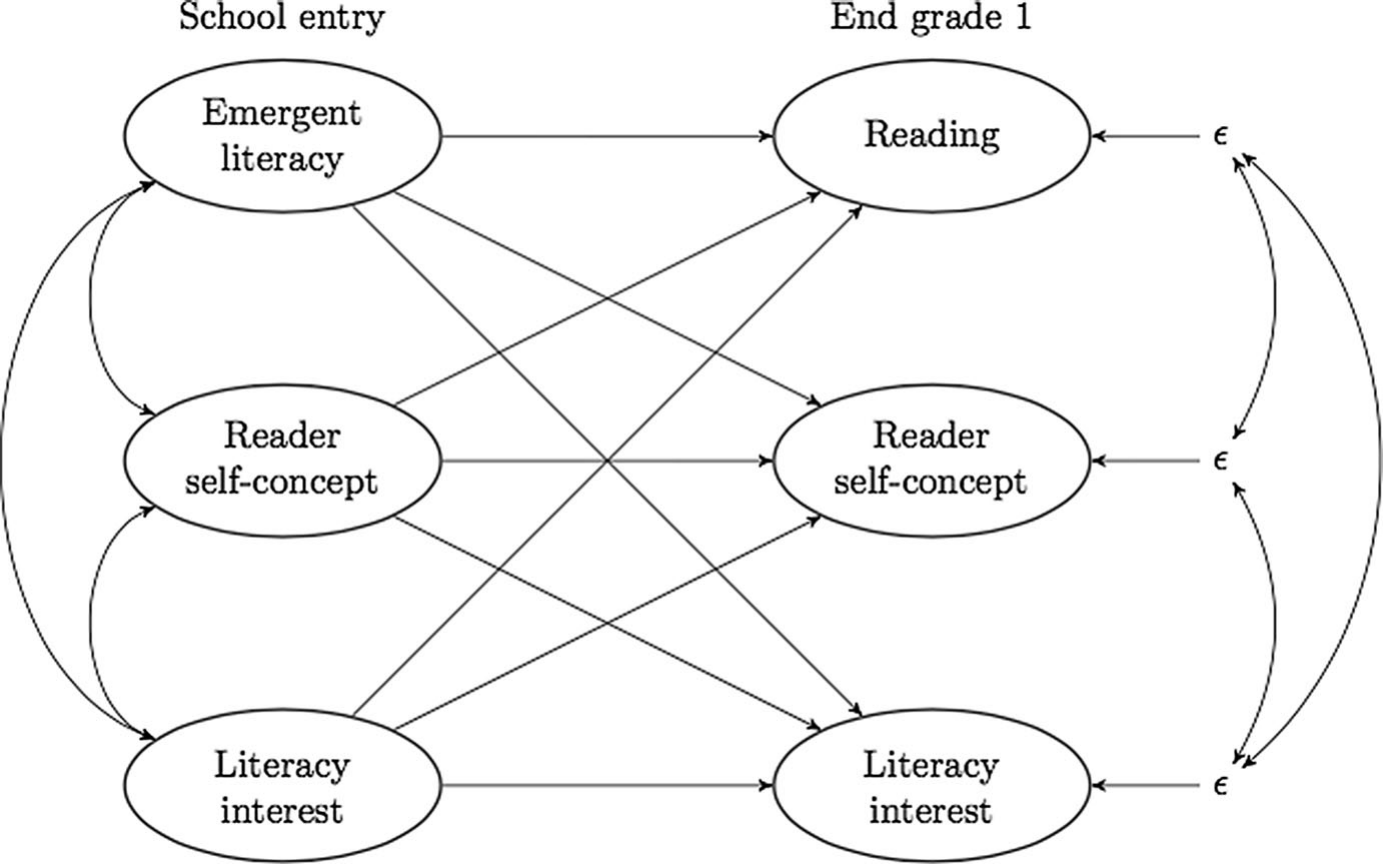
Conceptual model showing the cross-lagged relationships investigated in the present study

In the present study we employ a cross-lagged latent-variable model in a large sample of first-graders (see Fig. 2). The greater flexibility and complexity of this model, compared with the models shown in the left-hand rectangle in Fig. 1, may yield findings that also require us to adjust the ways in which we translate our understanding into language—so that we use terms and metaphors that target change and flexibility, rather than correlations at precise time points. In the present study, we have explored the stability of, and the cross-lagged relationships between, interest, reader self-concept and early reading skills as measured at school entry and at the end of the first year of formal schooling.

By doing this, we sought to answer the following research questions:

(i) Do children’s reading skills, literacy interest and reading self-concept show stability during the first year of formal reading instruction?
(ii) Do children’s motivational beliefs, in the form of literacy interest and reading self-concept, predict performance in reading during the first year of formal reading instruction, or is it rather the case that reading performance predicts subsequent motivational beliefs?
(iii) Do children’s reading self-concept at school entry predict their subsequent literacy interest?

## Method

### Sample

The sample consisted of 1141 students participating in the On Track (*På sporet*) study—a longitudinal study investigating the effect of early reading interventions for children at risk of reading difficulties (Lundetræ, Solheim, Schwippert and Uppstad, 2017). The children belonged to 62 classes (groups of students taught together) at 19 urban schools. The overall rate of participation was 97.7%, meaning that the sample is strongly representative of the target population.

Data were collected at two time points. At the first time point (T1), at school entry (August/September in the first grade), the participants’ age ranged from 5 to 6 years (68–79 months). At the second time point (T2; April/May in the first grade), their age ranged from 6 to 7 years (76–88 months). On both occasions, the children were tested for (emergent) literacy, reader self-concept and literacy interest.

In our analyses, we controlled for three background variables: gender, language and maternal educational level. Girls made up 50.8% of the sample. When it comes to language background, 15.4% of the students came from homes where both parents spoke non-Scandinavian languages. Of all students, 35.5% had a mother whose educational level was below university level.

### Measures and procedures

We realized that the students’ reading skills would improve considerably between the two time points at which we were going to test them. Hence it would not have been possible to use the same test on both occasions. To measure “emergent literacy” at T1, we used measures of phonological awareness and letter knowledge. At the end of the first grade, we used measures of word reading and sentence reading to measure “reading skills”. The measures of literacy interest and reader self-concept were also altered, albeit not as radically. At T1, the interest and self-concept items focused on situations that children are likely to experience before formal reading instruction. At T2, they instead related to experiences with independent reading in school and at home. For reliability estimation we used Guttman’s *λ*^2^ to avoid concerns with Cronbach’s alpha and also because in samples of the size in question, *λ*^2^ reduces the amount of bias in the reliability estimate (Revelle & Zinbarg, 2009; Sijtsma, 2009; Zinbarg, Revelle, Yovel & Li, 2005).

The participating students were tested individually at their respective schools during a period of 3 weeks. All 18 testers were experts in the field of reading instruction and individual testing. Prior to data collection, all testers received 6 h of training in administering this specific test battery. All tests were administered using a Lenovo Yoga Tablet 10 running Android 4.2. The students’ responses were scored and automatically recorded on the tablets.

### Measures of emergent literacy at T1

We created a second-order latent variable which consisted of measures of letter-sound knowledge and phonological awareness (operationalized as phoneme isolation and phoneme blending).

#### Letter-sound knowledge

We measured the students’ letter-sound knowledge using a matching test. Each item on the test started with the auditory presentation of a letter sound. Then the student was asked to indicate which one of four letters appearing on the screen matched that sound. We used upper-case letters because we assumed they would be more familiar to the students at school entry. Guttman’s *λ*^2^ for the 15 items included in the letter-sound measure was .85.

#### Phonological awareness

The test included two sub-tests intended to measure phonological awareness: phoneme isolation and phoneme blending. Both sub-tests consisted of items of increasing difficulty and were terminated after two sequential errors. This represents a compromise between the need for precise information and ethical considerations, and it means that the duration of these sub-tests differed across students, which may have influenced the reliability estimation because the students with the poorest performance obtained the same score (zero) on the last items of the sub-tests—in addition to a probably artificially high alpha.

The phoneme-isolation tasks measured the ability to identify the first sound in an auditorily presented word. The students were asked to isolate and pronounce the first sound of eight monosyllabic words representing common objects. There were two demonstration tasks. The first task used the following script: “In the picture you can see a dog. The very first sound of the word *dog* is *d*. Can you say *dog*? What is the first sound of *dog*?” In the second demonstration task, the tester named an object and then asked the student to say the first sound of the word, using the following script, “In the picture you can see a cat. What is the first sound of *cat*?” Corrective feedback was given during the demonstration tasks. Once the student performed the demonstration tasks correctly, the actual test began. Testers used the same script as in the second demonstration task but refrained from providing any corrective feedback. Guttman’s *λ*^2^ for the eight items of the phoneme-isolation task was .94.

The second phonological-awareness task included in the test battery was a forced-choice task measuring phoneme blending. The children were required to combine phonemes, presented to them auditorily and in correct order (e.g., /b/ /i/ /l/), to assemble a word, *bil* (meaning “car” in Norwegian). In order to ensure equal time lags between the sounds presented, the phoneme sequences were pre-recorded on the tablets so that each phoneme sequence was presented in an identical way to all the children. Guttman’s *λ*^2^ for the eight phoneme-blending items was .87.

### Measures of reading skill at T2

A second-order reading-skill factor was created from one measure of word reading and two measures of sentence reading.

#### Word reading

The word-reading tasks consisted of ten items. Each item consisted of a picture followed by four visually similar words, of which one corresponded to the picture. The child was asked to read the words as fast as possible and to check the word that matched the picture. Guttman’s *λ*^2^ for the ten word-reading items was .73.

#### Sentence reading

The first sentence-reading task consisted of ten items where a written sentence was followed by four pictures. The child was asked to choose the picture that corresponded best to the meaning of the sentence. Two demonstration tasks were conducted. Guttman’s *λ*^2^ for the ten items was .82.

The second sentence-reading task consisted of a picture and ten sentences. With each sentence, the child was instructed to find different objects in the picture (e.g., “Put a cross on the smallest window”). We conducted two demonstration tasks. Guttman’s *λ*^2^ for the ten items was .63.

### Measures of literacy interest and reader self-concept at T1

For literacy interest and reader self-concept at T1, we developed a self-report measure based on the Pictorial Scale of Perceived Competence (McTigue, Solheim, Walgermo, Foldnes, & Frijters, 2018). In this format, children receive picture support to understand the items, and they respond using two binary choices (Harter, 1982). For each item, the computer screen was first divided into two parts. The left-hand side showed a picture of a child who is engaged in a reading activity. On the right-hand side, a happy face and a sad face were shown next to each other. The tester orally presented the following script, “This girl [or boy, as appropriate] likes to visit the library [pointing at the happy face]. This girl does not like to visit the library [pointing at the sad face]. Which girl is more like you?” The student then chose one of the faces by touching the screen. If the student chose the happy face, the unhappy face disappeared and two circles—one small and one large—appeared below the happy face, whereupon the tester presented the following script, “Do you like to visit the library a lot [pointing at the large circle] or just a little bit [pointing at the small circle]?”

Alternatively, if the student chose the sad face, the happy face disappeared and he or she was asked, “Do you think visiting the library is very boring [tester pointing at the large circle] or just a little bit boring [tester pointing at the small circle]?” The student answered these questions by touching either the big or the small circle located below the chosen face. The possible student responses represented two binary decisions, yielding the following response scale ranging from 4 to 1: 4 = happy face, large circle; 3 = happy face, small circle; 2 = sad face, small circle; 1 = sad face, large circle.

The literacy-interest items relate to literacy situations and are not specifically linked to interest in letters. When designing the self-concept items, we aimed to link them to students’ expectations of how easy or difficult they thought it would be to learn how to read and write, i.e., perceived difficulty, and to how they compared themselves with their classmates. See Table 1 for all items included in the literacy-interest and reader self-concept scales. Items of interest and self-concept appeared in random order during the test. The sample-specific reliability of the literacy-interest scale as measured using Guttman’s *λ*^2^ was .67. Guttman’s *λ*^2^ for the five self-concept items was .62. The reliability of the present study is within the range given by Harter and Pike (1984) for their subscale for preschool and kindergarten children (.62–.83, *N* = 146).

**Table 1:** Items included in the literacy-interest and reader self-concept scales at T1 and T2

T1	T2
*Literacy interest*	
Do you like to look in and turn over pages in books?	Do you like to read?
Do you like to visit the library?	Do you think reading is boring?
Do you like it when someone reads to you at home?	Do you look forward to reading?
Do you like to receive a book as a present?	Do you like reading at home?
Do you like to look in books with a friend?	Do you think it is fun to read books?
Do you like it when the teacher reads aloud to the class?	
*Reader self*-*concept*	
Do you find learning the letters to be easy/difficult?	Do you find it easy to read books that you have chosen yourself?
Do you find learning to read to be easy/difficult?	Can you figure out hard words in a book even if there are no pictures?
Do you know as many letters as your classmates?	Do you think you are a good reader?
	Can you figure out hard words by yourself?
	Are you good at understanding the meaning of the words that you read?
	Are you a worse reader than many others in your class?

### Measures of literacy interest and reader self-concept at T2

When the students’ literacy interest and reader self-concept were measured at T2, they still responded by making two binary choices, but this time they did not receive any picture support. For example, on the item “Do you like to read? Yes or no,” the tester would record the student’s answer to that initial question and go on to ask either, if “Yes”, “Do you enjoy reading a lot or just a little bit?” or, if “No”, “Do you think reading is very boring or just a little bit boring?” The possible responses from each student again represent two binary decisions, yielding the following response scale ranging from 4 to 1: 4 = enjoys a lot, 3 = enjoys a little bit, 2 = just a little boring, and 1 = very boring. The tester read the items aloud and registered students’ responses on the tablet computer. Prior to the questions, the students were reassured that their teachers and parents would not be informed of their answers and that it was important that they answered the questions as honestly as possible.

The literacy-interest items focused on students’ feelings about reading in school and at home, whereas the self-concept items related to perceived competence in reading. Items of interest and self-concept appeared in random order during the test. When it comes to reliability, the literacy-interest measure had a Guttman’s *λ*^2^ of .85 and the self-concept measure had a Guttman’s *λ*^2^ of .61.

### Analytic strategy

For data analysis and latent-variable standard, we used the “lavaan” R package, version 0.4-9 (Rosseel, 2011). Given that all variables were binary or ordinal, estimation was performed using a diagonally weighted least-squares procedure, which takes into account the categorical nature of the data. Model fit was subsequently obtained using adjusted test statistics, whose asymptotic sampling distribution had a mean and a variance equal to those of the nominal Chi square distribution.

## Results

### Intraclass correlations

Because our sample is clustered in classes, we first investigated whether this clustering had to be incorporated into our model. Specifically, the presence of substantial intraclass correlation (ICC) for any of the sum scores associated with our six constructs would have suggested that a multilevel standard framework should be adopted. However, the ICCs associated with literacy interest, reader self-concept and emergent literacy/reading skills were all quite low: .039, .001 and .055, respectively, at T1 and .019, .035 and .045, respectively, at T2. The fact that the ICCs at T2 were in general of the same order of magnitude as those observed at T1 (the cluster-formation occasion) suggests that clustering effects were minimal during the year. Hence we deemed a single-level model to be appropriate for the data at hand.

### Fit of the measurement instruments and their intercorrelations

Before embedding our constructs of literacy interest (LI), reader self-concept (RSC) and emergent literacy/reading skills in a cross-lagged model, we investigated the fit of each construct to its indicators in separate one-factor models^1^ Reader self-concept at T1 is identified by only three indicators. The model is exactly identified and thus, trivially, has perfect fit. The root mean square error of approximation for each of the remaining five instruments is below .03, which is conventionally deemed to correspond to a good fit. In particular, literacy interest at T1, literacy interest at T2 and reader self-concept at T2 all have confidence intervals that contain zero, which represents a perfect fit. In Table 2, we present other common indexes of fit, whose values further support the claim that the proposed measurement instruments have a good fit to the data. Descriptive statistics for the composite variables of reader self-concept and literacy interest is given in Table 3.

**Table 2:** Approximate fit indexes for the six latent constructs

Construct	*df*	χ^2^	*p* value	RMSEA	RMSEA low	RMSEA high	TLI	CFI	SRMR
R1	248	396.9	.00	.023	.019	.027	.996	.996	.047
LI1	8	13.9	.09	.025	.000	.047	.994	.997	.022
RSC1	0	0		1.000	1.000	.000			
R2	374	767.1	.00	.030	.027	.033	.986	.987	.052
LI2	6	8.6	.20	.019	.000	.046	.999	1.000	.011
RSC2	5	8.5	.13	.025	.000	.053	.991	.996	.019

*df*, Degrees of freedom; *χ*^2^, Chi square; RMSEA, root mean square error of approximation; RMSEA low and high yield the 95% confidence interval for RMSEA; TLI, Tucker–Lewis index; CFI, comparative fit index; SRMR, standardized root mean square residual; R1, emergent literacy; LI, literacy interest; RSC, reader self-concept; R2, reading

**Table 3:** Descriptive statistics for the composite variables of reader self-concept and literacy interest

Variable	Mean	SD	Min/max
T1: school entry			
Reader self-concept composite	9.34	2.10	3/12
Literacy interest composite	21.69	2.49	11/24
T2: end of the first grade			
Reader self-concept composite	16.00	2.59	6/20
Literacy interest composite	20.76	3.43	6/24

Having established the soundness of our measurement models, we proceeded to estimate the correlations among the latent constructs, which are given in Table 4.

**Table 4:** Correlation matrix for latent constructs

	R1	RSC1	LI1	R2	RSC2	LI2
R1	1.00					
RSC1	.32	1.00				
LI1	−.09	.42	1.00			
R2	.70	.16	.00 n.s.	1.00		
RSC2	.19	.42	.19	.32	1.00	
LI2	.05 n.s.	.34	.46	.14	.62	1.00

All correlations are statistically significant at the .05 level, except those marked by “n.s.” R1, emergent literacy; LI, literacy interest; RSC, reader self-concept; R2, reading

### Estimating the cross-lagged model

**Fig. 3:**
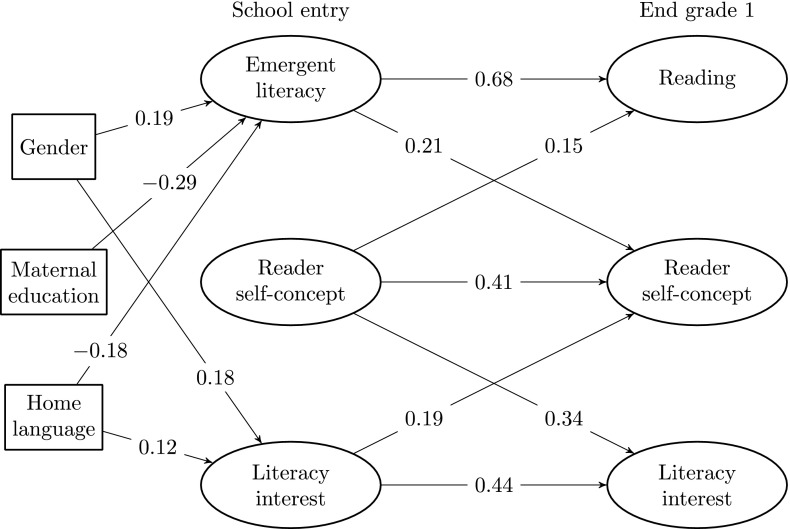
Panel model with standardized regression coefficients. The absence of paths indicates a lack of significance at the .10 level. Gender: 0 = boy, 1 = girl. Maternal education: 0 = higher (i.e., university/college) education, 1 = no higher education. Home language: 0 = both parents speak a non-scandinavian language, 1 = at least one parent speaks a Scandinavian language

Once we had established the soundness of the measurement instruments for emergent literacy/reading skills, reader self-concept and literacy interest on both measurement occasions, the next step was to estimate the cross-lagged model (shown in Fig. 3) and to add gender, language spoken at home and maternal educational level as covariates. The model fit test statistic was *χ*^2^ (*df* = 2740) = 4078 with an associated *p* value of 0. It is of course implausible that the suggested model should have a perfect fit to the underlying processes that we aim to understand in the present paper. The fact that our model will not attain a perfect description of the complex dynamics, combined with the large sample size, results in a highly significant rejection of the hypothesis of perfect model specification—as indicated by the *χ*^2^ statistic. However, the following fit indexes indicate an adequate fit of the model to the data: RMSEA = .021, CFI = .975, TLI = .974 and SRMR = .063. Therefore we consider the proposed model as a useful approximation of the underlying true developmental dynamics.

Finally, we estimated the cross-lagged model outlined in Fig. 1 with the addition of three background variables. Standardized estimates of stability and cross-relationships are given in Fig. 3, where we have omitted insignificant paths.

### Summary of results

We found all latent constructs to have an adequate fit to the data, allowing a meaningful estimation of their intercorrelations. With regard to our first research question, the results showed strong stability in children’s reading skills (.68) and moderate stability in their reader self-concept (.41) and literacy interest (.44) in the first grade. While none of the covariates predicted reader self-concept at school entry, we found that girls were significantly more interested in literacy than boys. Also, students whose parents did not speak Scandinavian were significantly less interested in literacy than students with at least one parent speaking Scandinavian. All three covariates were significantly related to emergent literacy, with girls having higher emergent literacy than boys, students with at least one Scandinavian-speaking parent having higher emergent literacy than students without a Scandinavian-speaking parent, and students with a higher level of maternal education having a higher level of emergent literacy. Concerning our second research question, we found bidirectional relationships between (emergent) reading skills and reader self-concept, but not between (emergent) reading skills and literacy interest. Finally, with regard to our third research question, we found an effect of reader self-concept on literacy interest, but also a bidirectional relationship between these two constructs within the first year of formal reading instruction.

## Discussion

In this study, we investigated the stability of, and cross-lagged relationships between, emergent literacy/reading skills, literacy interest and reader self-concept over the first year of school in a large sample of Norwegian 6- and 7-year-olds. In this section, we will first discuss the stability of the constructs within the first grade. Then we will discuss the cross-lagged associations between reading skills and the two motivational constructs of literacy interest and reader self-concept, followed by a discussion about the cross-lagged associations between the latter two constructs. Finally, we will examine the issue of cross-sectional correlations versus cross-lagged effects, and we will conclude with some reflections on how perceiving reading skills and motivation as *potentials* may contribute to a better understanding of their interrelationship.

### Stability of constructs across the first grade

The results of the present study show high stability between emergent literacy at school entry and reading skills by the end of the first grade. This finding is in line with previous studies that report stability in prereading and reading skills in preschool (Viljaranta et al., 2009), in preschool and the first grade (Lerkkanen, Rasku-Puttonen, Aunola & Nurmi, 2004), in the first grade (Chapman & Tunmer, 1997; Onatsu-Arvilommi & Nurmi, 2000) and in the early school years (Aunola, Nurmi, Niemi, Lerkkanen & Rasku-Puttonen, 2002; Schiefele et al., 2016). Our results thus confirm that the emergent literacy skills that children possess when they enter school are highly predictive of their future reading skills.

Literacy interest and reader self-beliefs are shaped by previous experiences (e.g. Bandura, 1995). In the present study, the motivational items at school entry reflected situations that the students could be expected to have participated in even if they had limited or no experience of learning to read. By the end of the first grade, the motivational items focused on the students’ experiences related to reading. For literacy interest, this meant a shift from participation in literacy-related activities (e.g., enjoys being read to or looking at books) to independent reading in school or at home (e.g., enjoys reading). For reader self-concept, it implied a shift from perceived expected difficulty in learning how to read to perceived competence in reading. Comparison with classmates was included at both time points. Despite these shifts in perspective, we observed moderate stability in both motivational-belief constructs in the first grade. These results, which are in line with Chapman and Tunmer’s (1997) reports of moderate stability in reader self-concept across the early school years, imply that Norwegian children’s *informal* literacy experiences before school entry (i.e., in the *barnehage* and at home) are associated with their literacy interest and reader self-concept by the end of the first grade. This is a noteworthy finding, given that Norwegian children go directly from the *barnehage* with its lack of formal reading and writing instruction and its largely child-driven activities to school proper. Because children who show an interest in letters and books in the *barnehage* are encouraged and supported to gradually gain more experiences with literacy, whereas those who are uninterested in literacy activities are free to choose other activities, there is huge variation in the (amount of) experience with literacy activities that Norwegian school starters bring with them to school.

### Cross-lagged associations between motivational constructs and reading performance

Literacy interest was found not to be related to emergent literacy at school entry nor to reading skills after one year of school. This might be in part because the measure used in the present study was not directly linked to learning to read. However, this finding is in line with those of studies that included such items (Viljaranta et al., 2009; Nurmi & Aunola, 2005) and still found no cross-lagged effects between interest in literacy-related tasks and literacy performance in preschool children and first-graders. Viljaranta et al. (2009) explain their finding with reference to the regular nature of Finnish orthography and the fact that most Finnish children read accurately by the end of the first grade, but they hypothesize that interest in literacy-related tasks will be of greater relevance later on, when more demanding literacy tasks such as reading fluency and text comprehension are introduced. In line with this reasoning, Schiefele et al. (2016) reported that intrinsic motivation was in fact associated with reading comprehension in the second and third grades.

By contrast, our analysis found statistically significant, reciprocal effects between reading skills and reader self-concept within the first grade. Unlike previously reported research, we found that emergent reading skills affected the formation of reader self-concept even during the first year of formal reading instruction. Our results also revealed that there was a small positive effect of a strong reader self-concept on subsequent reading skills in students at the same levels of interest and emergent literacy skill. This finding is in line with those reported for older students (see Murphy & Alexander, 2000; Zimmerman, 2000) and indicates that even young students who view themselves as competent are better at developing skills than students who exhibit poor self-belief and hence anticipate failure. Given the results of the present study, the question arises whether the practice prevalent in Norwegian *barnehager* of supporting those children who are already interested in letters and reading but letting the other children choose other activities may actually widen the gap between children who enter the *barnehage* with different predispositions and aptitudes (Stanovich, 2009).

### Cross-lagged associations between literacy interest and reader self-concept

The present study also revealed statistically significant bidirectional relationships between reader self-concept and literacy interest across the first grade. The strongest association was observed from reader self-concept onto interest in reading, which supports the suggestion that students’ self-concept of their ability provides a basis for how interested they are in an activity (Eccles, Wigfield, Harold & Blumenfeld, 1993). This finding is in line with those of previous studies that investigated associations between (i) motivation for literacy tasks and reader self-concept in first-graders (Nurmi & Aunola, 2005), (ii) general school-related motivation and self-beliefs in grades 1–4 (Spinath & Spinath, 2005) and (iii) domain-specific self-competence and values in grades 1–12 (Jacobs et al., 2002). We also found a small, but statistically significant, positive effect of literacy interest on reader self-concept. In a previous study carried out on the same sample of students, interest at school entry was found to moderate the relationship between the students’ reader self-concept and their emergent literacy skills except that, at high levels of interest, the significant relationship between reader self-concept and emergent literacy disappeared (Walgermo et al., 2018).

### Cross-sectional correlations versus cross-lagged effects

Some previous studies of first-graders have looked exclusively at cross-sectional correlations between motivational factors and early reading (Chapman & Tunmer, 1997; Morgan et al., 2008). In the present study, the correlation between emergent literacy/reading skills and reader self-concept was .32 at both time points, which is substantially higher than the values reported by Chapman and Tunmer (1997) and by Morgan et al. (2008) for the first 6 months of the first grade. Between emergent literacy/reading skills and literacy interest, we found correlations of −.09 at school entry and .14 at the end of the first grade. A cross-sectional correlation of .32 between skills and self-concept, studied in isolation, indicates a medium to strong relationship between these two constructs during the first grade. However, the cross-lagged model used in the present study shows that motivational factors actually explain only a small (albeit statistically significant) amount of the variance in reading skills when students’ initial level of emergent literacy is controlled for and when relevant covariates are taken into account. This finding suggests that previous studies that used a cross-sectional design may have overestimated the effect of motivational factors on early reading development.

### The dynamics of reading skills and motivation in terms of potentiality

In the above sections, we have shown how a cross-lagged model, compared with sectional and cross-sectional ones, may add more flexibility and complexity to our understanding of the dynamics of reading skills and motivation over time. In this last section, we will discuss how the improved comprehension thus gained of the dynamics of reading skills and motivation can be seen from a new perspective.

Reading research taking the cognitive approach has tended to describe reading development using flow charts, analogously to the workings of a computer (e.g., Hergenhahn, 1986, p. 543)—generally as a modular and rather isolated process. Tønnessen and Uppstad (2015, p. 24) claim that this kind of modular thinking (cf. Fodor, 1983; von Eckardt, 1993) makes it difficult to find a connection between learning on the one hand and motivation, stimulation and reward on the other. In line with Tønnessen and Uppstad, we consider that modular perspectives have been overused in this field and that insufficient focus has therefore been placed on changes in interrelationships. The present study, with its particular time window, sheds some light on the dynamic nature of the interplay between motivation and skills. In our opinion, Tønnessen’s idea of seeing reading skills as a *potential* (Tønnessen, 2011) offers a constructive theoretical framework for conceptualizing the interplay between reading and motivational constructs. According to Tønnessen and Uppstad (2015), the development of reading skills is deeply influenced by adjoining environmental factors and individual characteristics such as motivational constructs. In their view, *both* reading skills and motivation are “potentialities” that will be realized by individuals to a different extent and in different ways. However, motivation is not a skill as such; it develops in tandem with the skill to which it is linked—sometimes at a different pace or in a different direction. One characteristic of their mutual relationship is that in order to realize one’s full potential as a reader, one should have a level of self-belief that slightly exceeds one’s actual level of reading skill (Bandura, 1995; Frankl, 1985).

On the strength of our new cross-lagged data, relating to the nature of reading development as a complex human process, we believe there is a need for a more apposite way of conceiving the dynamic interrelationship between the constructs involved. If we consider reading skills, literacy interest and reader self-concept as potentialities, then we may conceive of the dynamics between them in ecological terms by comparing them to organisms living in a dynamic, symbiotic relationship. Organisms living in symbiosis tend to derive mutual benefit from this arrangement. They all develop, although not strictly in parallel to one another, and whatever one of them does will affect the others. The fact that the results from the present study portray a constellation of reading skills and motivational constructs that differs from that found for older students (Harlaar, Deater-Deckard, Thompson, DeThorne, & Petrill, 2011) exemplifies the dynamic, symbiotic nature of the motivation–skills relationship: the interrelationship between these constructs seems to change as children gain more experience with reading situations and as the tasks they are given grow more complex.

## Conclusion

The present study has found evidence of significant bidirectional relationships between reader self-concept and early reading skills and between literacy interest and reader self-concept within the first year of formal schooling. In sum, these findings suggest that relationships between self-concept and early reading skills start affecting children’s reading development even before formal reading instruction begins. Literacy interest was not found to be related to early reading at this stage, but previous research has found that the development of reading skills, interest in reading and reader self-concept in older students is interrelated: students who show a well-developed literacy interest, slightly higher than their corresponding level of skill, and who perceive themselves to be competent and able readers will more often take on challenging reading tasks and are more likely to realize a greater part of their reader potential than those who have less positive self-concepts (Bandura, 1995; Frankl, 1985). By contrast, previous research has found that realistic or low levels of self-belief tend to have a negative effect on personal improvement in general.

Against this background, increased use of cross-lagged designs in future studies will probably yield insights into reading development that will help us to better understand the symbiotic relationship between reading skills and motivation. To ensure that motivation and reading skills can influence each other in mutually beneficial ways, we need to know what levels of interest and reader self-concept are optimal for children’s reading development. At this time, we can only say that taking an interest in literacy and having a robust reader self-concept seem to be desirable personal characteristics that, in a symbiotic relationship with reading skills, can facilitate the realization of students’ full potential as readers.

## Limitations of the present study

Several of the limitations of the present study are linked to the nature of the measures used. Given that the students’ reading competence was bound to develop considerably over the period studied as a consequence of the formal reading instruction they would receive, we were not able to use identical measures to test either their reading performance or their motivation at the two time points. At T1 we measured levels of emergent literacy and interest in literacy, while at T2 we measured reading skills and interest in reading. For reader self-concept, the items shifted between T1 and T2 from perceived difficulty of the process of learning how to read to perceived reading competence based on experiences from learning how to read over the first year in school.

Measuring the reading motivation of students on the verge of formal reading instruction also means that the amount of experiences on which the students could base their answers would differ a great deal. Some students gave their answers based on a variety of experiences with literacy from kindergarten and home, whereas others had very few experiences to which they could relate their answers. However, the stability of interest as measured across the first year of formal instruction does not indicate any major statistical weaknesses.

Finally, one important clarification that must be made is that while our cross-lagged model shows bidirectional relationships, we do not claim that there are any *causal* relationships between the factors in question. Even though the covariates, which are based on sound theoretical assumptions, are controlled for, there may still be underlying extraneous factors.

## Footnotes

1 Prior to the present investigation, we carried out latent-variable mean tests which showed that the interventions in the at-risk group had no effect on children’s motivation for reading. Hence we chose to include all participating students in the sample for the present study.
